# Rice quality and its impacts on food security and sustainability in Bangladesh

**DOI:** 10.1371/journal.pone.0261118

**Published:** 2021-12-31

**Authors:** Indrani Saha, Alvaro Durand-Morat, Lawton Lanier Nalley, Mohammad Jahangir Alam, Rodolfo Nayga

**Affiliations:** 1 Dept. of Agricultural Economics and Agribusiness. University of Arkansas, Fayetteville, Arkansas, United States of America; 2 Dept. of Agribusiness and Marketing, Bangladesh Agricultural University, Mymensingh, Bangladesh; 3 Department of Agricultural Economics. Texas A&M University, College Station, Texas, United States of America; Bangabandhu Sheikh Mujibur Rahman Agricultural University, BANGLADESH

## Abstract

Rice market efficiency is important for food security in countries where rice is a staple. We assess the impact of rice quality on rice prices, food security, and environmental sustainability in Bangladesh. We find that while price varies as expected for most quality attributes, it is unaffected by a broken percentage below 24.9 percent. This reveals a potential inefficiency, considering the average 5 percent broken rate observed in the market. An increase in the broken rate of milled rice within the limits supported by our findings can, *ceteris paribus*, increase rice rations by 4.66 million a year, or conversely, yield the current number of rice rations using 170.79 thousand fewer hectares and cutting emissions by 1.48 million metric tons of CO2 equivalent. Thus, producing rice based on quality assessment can improve food security and its sustainability.

## Introduction

Rice (*Oryza sativa* L.) is the main food for over half of the world population [1]. In order to feed 10 billion people in 2050, it is imperative to consider the sustainability of food systems, including better utilizing food already produced [2]. In the majority of global rice markets, broken rice, a sub-product of rice milling, is considered of inferior quality compared to milled rice despite having the same nutritional value, and thus is sometimes funneled into uses other than human consumption. This allocation of broken rice into non-human consumption can dampen food security, particularly in Asia, which accounts for 90 percent of global rice production. Moreover, the increasing use of broken rice as a feed rather than food [3] undermines the sustainability of food production, primarily the water footprint of agriculture, since rice uses water more intensively than other feed grains such as corn [4].

Because rice is a field-to-plate crop, unlike wheat, maize and soybeans which are typically processed, visual attributes can often drive demand and price. Understanding the market value of different rice attributes is important for the development of a more competitive domestic rice supply chain [5]. First, domestic rice-breeding programs can focus on varietal attributes that would be appealing for domestic consumers, and investments in rice milling and handling can be prioritized according to the potential economic market return. Second, understanding the behavior of rice markets, and more specifically how the value of rice varies with certain quality attributes, can also help identify potential market inefficiencies and support the development of policies, such as commercialization standards, aimed at improving consumer and producer welfare.

A better understanding of consumer preferences for rice quality attributes, especially broken rice, can help cater rice specifically for different market segments via different quality presentations and price options, thus potentially increasing the use of rice for food rather than feed. The objective of this study is to assess the effect of rice quality, focusing on broken rice, on rice prices, and analyze its implications on food security and environmental sustainability. Our analysis focuses on the rice market in Bangladesh, one of the largest rice markets in the world. Bangladesh has made significant progress in improving the livelihood of its population in the last two decades. Poverty and extreme poverty decreased from 49 percent in 2000 to 13 percent in 2016 [6], undernourishment decreased from 21 percent in 2000 to 15 percent in 2017, and stunting among children decreased from 51 percent in 2000 to 36 percent in 2014 [8]. Despite this progress, 30.5 percent of the population still suffered from moderate food insecurity and 10 percent from extreme food insecurity in 2017, while 33 percent children were classified as underweight in 2014 [7]. More recent estimates show that poverty and extreme poverty increased sharply to 42 percent and 28.5 percent in December 2020 due to the Covid-19 pandemic [8]. The crucial role of rice as a livelihood for most rural households and a staple food across the country highlights the importance of better understanding of how the rice market in Bangladesh functions.

Although this study focuses particularly on Bangladesh, the implications of the findings are much broader, and highlight the potential food security and environmental benefits of preference-matching [9], that is, having consumer-driven markets that supply the preferred products at competitive prices.

### Rice market in Bangladesh

The rice industry in Bangladesh represents 70 percent of the agricultural gross domestic product, and is the primary income source for over 48 percent of the rural population [10]. Rice is the staple in Bangladesh; with an average per-capita consumption rate of 144.5 kg/year [11], it accounts for 67.5 percent of the daily caloric intake [12].

Bangladesh is the third largest rice market globally, producing and consuming 34.6 and 35.8 million metric tons (MMT) in 2020/2021 [13]. There are three main rice growing seasons. The Boro (winter) rice crop, planted between November and early January and harvested in the Spring, has the highest productivity, and accounts for around 40 percent of the planted area and 55 percent of the total volume of production. The Aus (pre Monsoon) rice crop, planted during the Spring and harvested in the Summer, is the smallest of the three, accounting for around 10 percent of the planted area and 7 percent of the total volume of production. Finally, the Aman (Monsoon) rice crop, planted in late Spring and Summer and harvested late in the Fall-early Winter, is the largest crop in terms of planted area (around 50 percent) and second largest in terms of volume of production (around 38 percent) [14].

Some 40 percent of the rice produced in Bangladesh is for home consumption and processed using primarily home-made tools such as Dhekis [15], or custom mills that process rice for a fee [16]. The rest of the rice is marketed through paddy traders to rice mills. The milling sector, which comprises around 17,000 rice mills, transforms the rice from paddy to either parboiled or non-parboiled milled rice. Most rice consumed in Bangladesh is parboiled, although non-parboiled rice is preferred in some areas, such as in the western Khulna Division [16]. Wholesalers aggregate rice from mills and sell to retailers mostly in 50-kg polyester bags. Most rice in Bangladesh is sold by specialized rice retailers in open markets, and purchased either in sealed pre-packed bags or loose. The public procurement system operates as a separate value chain, aimed at supporting the lower income households, and usually pays a premium for paddy rice, and is distributed via public programs and authorized retailers.

There rice market in Bangladesh is highly segmented, with different types of rice having distinct regional and socioeconomic markets and carrying notably different prices. One common classification includes fine rice (length/width ratio of more than 3), coarse (bold) rice (length/width ratio < 2.1), and aromatic rice (e.g., imported basmati and jasmine rice, or domestically produced Chinigura and Kalijira rice).

There is growing pressure to improve rice productivity in Bangladesh to cope with increases in rice demand and shrinking production resources (e.g., arable land). Rice productivity increased by around 1 percent annually from an average of 4.07 metric tons (MT) per hectare in 2010 to 4.51 MT/hectare in 2020 [14], in part thanks to the adoption of high-yielding inbred and hybrid varieties released by the International Rice Research Institute and the Bangladesh Rice Research Institute [17]. Total rice production grew at the same pace as productivity given that harvested rice land has remained stable in the last decade. Bangladesh is almost self-sufficient in rice (domestic production accounted for 97 percent of domestic consumption between 2010 and 2020), but it often relies on the international market when domestic production is disrupted, such as the significant crop loses experienced in marketing year 2017/18, which pushed rice imports up to 2.4 MMT [14].

### Literature review on rice quality preferences

Globally, consumer preferences for rice are heterogenous, and therefore the value attached to specific attributes varies geographically and by the socio-cultural context in which rice consumption is embedded. There is growing evidence, primarily from Asia and Africa, that indicates consumers are increasingly aware of rice quality, even amongst low-income households [5, 18–23]. Consumers in Bangladesh and the eastern side of India consider taste, whiteness, slenderness, short grain, and aroma as attributes associated with high quality rice [24]. Good taste, whiteness, and slenderness were consistently mentioned in the top three most important rice characteristics preferred by urban households in Dhaka, and by most households across other urban areas and socio-economic classes [16]. Parboiled rice (both double-boiled and single-boiled) is popular across Bangladesh, and comes in two forms: (a) double-boiled rice refers to a modified parboiling process in which the paddy rice is (1) stemed, (2) soaked, (3) stemed a second time, and (4) dried; and (b) single-boiled rice refers to the traditional steps in the parboiling process, in which paddy rice is (1) soaked, (2) stemed, and (3) dried.

Short and bold grain rice are considered fine quality rice in the southern parts of India (e.g., Tamil Nadu, Kerela) and Sri Lanka [25], while consumers in the northern and western part of India and Pakistan prefer slender, long-grain rice [26]. Medium size slender rice dominates in Bangladesh and Indonesia, long-grain rice is the most popular in the rest of southeast Asia, and short and bold rice is strongly preferred in northeast Asia [5]. In the Philippines and Bangladesh, broken rice is perceived as a low quality product, and therefore the market price of rice decreases as the percentage of broken rice kernels increases [18, 25]. Households in Malaysia mostly prefer white long-grain aromatic rice, while in Indonesia, consumers prefer rice varieties that are long, slender in shape and with a white belly [25]. Rice that cooks firm and dry is widely preferred by South Asian consumers because combining these two attributes characterizes parboiled rice, which is traditionally consumed in many regions in Bangladesh [27]. Rice consumers from southeast Asia (Thailand, Philippines, Vietnam, Cambodia) mostly prefer rice with a sticky texture (low amylose content) and fragrance [23], while consumers in the northern and western part of India and Pakistan prefer nonsticky and aromatic rice [26]. There are more consumers in Bangladesh that prefer aromatic rice than those who regularly consume it, possibly due to the higher price and delicacy perception of aromatic rice [16].

## Materials and methods

### Data

The data used in this study consists of 300 milled rice samples collected from ten different retail rice markets across urban Dhaka, Bangladesh, in September 2020. To capture potential price and quality heterogeneity due to differences in household socioeconomic status (e.g., income levels), the markets were selected from different neighborhoods of Dhaka, which were in turn chosen based on the average poverty headcount ratio of each respective neighborhood as estimated by the World Bank [28]. The markets in Dhanmondi, Gulshan, and Mohammadpur Town Hall are located in high-income neighborhoods, as reflected by their low poverty headcount of 1.3, 3.3, and 4 percent, respectively. Farmgate/Tejgaon, Mirpur, and Agargaon Taltola are middle-income markets, with a poverty headcount ratio of 6.7, 6.6, and 7.6 percent, respectively. Finally, the Thathari Bazar/Old Dhaka, Jatrabari, Adabor, and Khilgaon markets are classified as low-income markets and located in neighborhoods with a poverty headcount ratio above 10 percent. There are many different rice types sold across Dhaka, including premium fragrant rice such as domestic Chinigura and Kalijira rice, and imported jasmine and basmati rice. Aromatic rice usually sells at a premium because of its fragrance; however, since fragrance is not one of the attributes measured in this study, we focused on long- and medium-grain, non-fragrant rice, the most popular rice types sold in Bangladesh. We collected an average of 5 1-kilogram rice samples from 60 different vendors (at least 5 vendors from each market), and recorded their listed retail market price, origin (domestic or imported), and whether the rice was parboiled and, if so, whether single or double parboiled.

### Measurement of physical attributes of rice

The rice samples were processed to ascertain their quality considering the following intrinsic quality attributes: broken percentage, chalk percentage, color, length, width, homogeneity (e.g., long-grain, medium-grain, short-grain, mixed grains), and parboiling. The Vibe QM3 Rice Analyzer was used to obtain quality attributes and was calibrated to the Bangladeshi standard for milled rice (Supplementary file A) developed by the Bangladesh Standards and Testing Institution [29].

Milled rice is composed of head rice, defined by the Bangladeshi rice standard as a kernel whose length is at least 8/10 of the average length of the corresponding whole kernel, and broken rice, defined as a kernel whose length is less than 8/10 of the average length of the corresponding whole kernel [29]. The variable *Broken percentage* is defined as:

$$\begin{array}{c} Broken\;{percentage}_{i}=\frac{{WB}_{i}}{\left({WB}_{i}+{WH}_{i}\right)}*100 \end{array}$$ (i)

where *WB*_*i*_ and *WH*_*i*_ are the weight of broken and whole rice in sample *i*, respectively.

The chalk percentage is defined as the weight of rice kernels with half or more of their area opaque and chalky relative to the weight of the sample [30]. The variable *Chalk percentage* is defined as:

$$\begin{array}{c} Chalk\;{percentage}_{i}=\frac{{WC}_{i}}{Weight\;of\;Working\;Sample\;i}*100 \end{array}$$ (ii)

where *WC*_*i*_ is the weight of chalk rice in sample *i*.

Color is measured in the CIELAB color space. CIELAB is made up of three channels: L* represents the lightness value of the color; a* represents green or red respectively; and b* measures blue as negative and yellow as positive value. *Color* is estimated as:

$$\begin{array}{c} {Color}_{i}=\sqrt{L_{i}^{2}+a_{i}^{2}+b_{i}^{2}} \end{array}$$ (iii)

The higher the value of color, the whiter the rice sample.

The size of rice is measured by the length and width of the kernels (in millimeters), and consequently variables *Length* and *Width* represent the average length and width across all kernels *n* in sample *i*.

Finally, the shape of rice in sample *i* is measured by the length to width ratio (*LWR*) as follows:

$$\begin{array}{c} {LWR}_{i}={Length}_{i}/{Width}_{i} \end{array}$$ (iv)

Based on the LWR, the Bangladesh rice standard distinguishes between slender (> 2.8), medium (2.1–2.7), and coarse/round (< 2.1) rice (S1 File).

Rice homogeneity refers to whether the rice sample can be classified as of one type (e.g., long-grain or medium-grain rice), or a mix of different types. We adopted USDA’s definition of milled rice classes [31], according to which milled rice can be classified as of one type (e.g., long grain) if it contains no more than 10.0 percent of whole or broken kernels of other type (e.g., medium or short grain rice). The variable *Homogeneity* was specified a a binary variable equal to 1 if the sample was mixed, and zero if it was classified as either long-, medium-, or short-grain rice.

Finally, we created two dummy variables for parboiling, *Double-boil* and *Single-boil*, to identify whether the rice was single- and double-boiled rice. In Bangladesh, single-boiled rice is produced following the traditional three-step process of (1) soaking, (2) steaming, and (3) drying paddy rice, while double-boiled rice is steamed twice (once before and once after soaking) and then dried, and hence follows a four-step approach [32].

Vendors identified all the rice samples collected for this study as domestic rice, which is expected considering that Bangladesh was 97.2 percent rice self-sufficient from 2015–2020 [13].

We conducted a piecewise analysis to ascertain if the effect of an independent variable (e.g., broken percentage) on rice price varies over the observed ranges [33]. This is particularly important for the broken and chalk percentages, whose levels can be altered by production and processing practices to match the market demand.

### Pure hedonic price model

Pure Hedonic pricing regressions are based on Lancaster’s theory of value, which suggests that any good can be described in terms of its attributes or characteristics [34]. Accordingly, the price consumers are willing to pay for rice is a function of physical characteristics of rice, defined as:

$$\begin{array}{c} P_{i\;}=\;\beta X_{i}+\;\varepsilon_{i} \end{array}$$ (v)

where *P*_*i*_ represents the price paid by consumer *i*, *X_i_* is a vector of rice quality attributes, and *ε*_*i*_ is the error term. The model was estimated with and without market fixed effects to account for differences across the markets surveyed. Based on the variables defined above, the econometric equation is defined as:

$${Price}_{i}=\beta_{0}+\;\beta_{1}\;{Broken\;percentage}_{i}+\beta_{2}\;Chalk\;{percentage}_{i}+\beta_{3}\;{Color}_{i}+\beta_{4}\;{LWR}_{i}+\beta_{5}\;{Homogeneity}_{i}+\beta_{6}\;{Double-boil}_{i}+\beta_{7}\;{Single-boil}_{i}+\sum_{j=1}^{9}\beta_{j}\;{Market}_{ij}+\varepsilon_{i}$$ (vi)

Two hedonic price models were estimated, Model 1 with no market fixed effects, and Model 2 with market fixed effects. The dependent variable *Price*, and the independent continuous variables *LWR* and *Color* are expressed in logarithm. We conducted a piecewise analysis for broken percentage (S2 File) to ascertain whether the correlation between broken percentage and price changes with the level of broken percentage. The results from the piecewise analysis highlight the existence of two distinctive segments, which led us to to split the broken percentage variable into the following two variables: *Broken percentage 1*, including observations with a broken percentage less than or equal to 24.94 percent, and zero otherwise; and *Broken percentage 2*, including observations with a broken percentage greater than 24.94 percent, and zero otherwise. Because of the presence of zero values, the variables *Broken percentage 1*, *Broken percentage 2*, and *Chalk percentage* are expressed in nominal terms. Finally, the variables *Homogeneity*, *Double-boil*, and *Single-boil* are binary variables.

Based on the literature on rice quality preferences in Bangladesh, we expect that variables *Broken percentage 1, Broken percentage 2*, and *Chalk percentage* have negative coefficients (e.g., negative correlation with market prices), while *Color*, *LWR*, *Double-boil*, and *Single-boil* have positive coefficients. We hypothesize that the variable *Homogeneity* is positively correlated with the market price of rice, and thus has a positive coefficient. Finally, we expect that, everything else equal, market prices would be negatively correlated with the income level in the selected markets.

## Results and discussion

### Physical characteristics of rice

Table 1 shows the descriptive statistics of the retail price and the selected rice quality attributes for all samples. The average retail price was 63.84 Bangladeshi Taka (BDT)/kg, with samples ranging from 44 to 100 BDT/kg. Of the total 300 rice samples analyzed, 209 were double-boiled, 45 single-boiled, and 46 non-boiled rice.

**Table 1:** Descriptive statistics of the retail price of rice and selected rice quality attributes for 300 milled rice samples.

Variable	Mean	Std. Dev.	Min.	Max.	Expected signs
Price (BDT/kg)†	63.84	11.32	44.00	100.00	
Broken percentage	5.35	7.07	0.43	46.15	-
Chalk percentage	12.56	22.13	0.00	89.56	-
Color	68.76	5.27	56.86	79.61	+
Length (mm)	5.07	0.55	3.75	6.20	
Width (mm)	1.98	0.29	1.56	2.63	
LWR	2.62	0.51	1.59	3.90	+

^†^. 1 USD = 84.787 BDT (average exchange rate in July 2021)

The broken percentage ranged from 0.43 percent to 46.15 percent, with an average of 5.35 percent. The average broken percentage is considered low by international trade standards, given that rice with 5 percent or less broken is typically classified as high-quality rice. Based on the Bangladeshi standard for milled rice [29], a 5 percent broken rate corresponds to high-quality rice (Grade I) for both non-parboiled and parboiled rice [29]. The low average broken percentage is explained in part by the fact that 84.7 percent of the samples are parboiled rice, which has been established to reduce the percentage of broken rice [35].

The chalk percentage ranged from 0 percent to 89.56 percent, with an average of 12.56 percent. The large variability is partially explain by the fact that our samples include parboiled rice, which has a low chalk rate as the parboiling process decreases chalkiness [36]. The average chalk rate of the samples corresponds to sub-standard rice according to the Bangladesh standard [29].

Color ranged from 56.86 to 79.61, with an average of 68.76. Regarding rice size and shape, the average length, width, and LWR were 5.07 mm, 1.98 mm, and 2.62, respectively, which according to the Bangladesh standard for milled rice (S1 File) corresponds to medium grain rice. The assessment of each sample by size and shape revealed that 95 samples correspond to medium grain, 16 to short grain, and 189 samples were considered mixes of different rice types. This highlights a potential inefficiency between what the market supplies and consumers prefer, given that short grain rice ranks among the top 5 quality attributes valued by urban Bangladeshi consumers [16, 24].

The price and broken percentage of milled rice vary significantly (p <0.01 and p<0.10, respectively) across markets (Table 2). The average rice price is the highest in the high-income markets of Dhanmondi (market 1), Gulshan (2), and Mohammadpur (3), while the low-income markets of Thathari Bazar/Old Dhaka (7), Jatrabari (8), Adabor (9), and Khilgaon (10), recorded the lowest prices. Milled rice purchased in Mohammadpur (market 3) have the lowest, and that from Jatrabari (8) have the highest, average broken percentage.

**Table 2:** Mean value of retail price and selected rice quality attributes by markets.

Variable	Markets (income level)*										P-Value^†^
	3 (high)	2 (high)	1 (high)	4 (mid)	5 (mid)	6 (mid)	9 (low)	8 (low)	10 (low)	7 (low)	
Price (BDT/kg)^‡^	72.77^a^	69.30^ab^	68.30 ^abc^	65.43^bc^	64.36 ^c^	63.66^c^	60.77 ^d^	59.47^d^	57.50 ^d^	56.87^d^	0.000***
Broken percentage^‡^	3.75 ^a^	4.53^cd^	5.42 ^e^	4.02^b^	6.25 ^h^	5.95^f^	4.06 ^c^	8.11^i^	6.03 ^g^	5.35^d^	0.096*
Chalk percentage	12.35	14.37	10.78	13.29	12.66	12.31	14.11	13.17	12.11	10.42	0.848
Color	69.67	69.23	68.80	68.95	68.90	67.39	68.89	69.35	68.20	68.21	0.825
Length (mm)	5.04	5.07	5.06	5.06	4.98	5.04	5.10	5.07	5.13	5.15	0.979
Width (mm)	1.92	1.93	1.93	1.99	1.97	1.97	1.96	2.05	1.99	2.06	0.217
LWR	2.70	2.71	2.70	2.61	2.59	2.61	2.64	2.53	2.61	2.53	0.867

* Market variables 1 through 10 correspond to the following neighborhoods (1) Dhanmondi (high income), (2) Gulshan (high income), (3) Mohammadpur (high income), (4) Farmgate/Tejgaon (middle income), (5) Mirpur (middle income), (6) Agargaon Taltola (middle income), (7) Thathari Bazar/Old Dhaka (low income), (8) Jatrabari (low income), (9) Adabor (low income), and (10) Khilgaon (low income).

^†^ For the P-value (Kruskal-Wallis test),

***, **, * represent 1%, 5%, and 10% significance level.

^‡^ Dunn’s test for difference in means across markets; different letters mean statistical difference across markets at the 5% level.

The results from the piecewise analysis for broken percentage (S2 File) highlight the existence of two distinctive segments, which led us to to split the broken percentage variable into the following two variables: *Broken percentage 1*, including observations with a broken percentage less than or equal to 24.94 percent, and zero otherwise; and *Broken percentage 2*, including observations with a broken percentage greater than 24.94 percent, and zero otherwise.

### Pure hedonic price model

Table 3 shows the results for the two hedonic price models. Both models were found not to be plagued with heteroscedasticity and collinearity according to the Breusch-Pagan test (chi2 = 0.55 (p >0.459) for Model 1 and 2.55 (p >0.110) for Model 2) and variance inflation factor tests (Mean vif = 1.82 for Model 1 and 1.85 for Model 2). Model 1 presents the best fit (highest F value) amongst the two models, which suggests that consumers value the selected rice quality attributes similarly regardless of market location. The magnitude and significance of the coefficients of the selected quality variables is similar between Model 1 and 2, which signifies the robustness of the findings across model specficiations.

**Table 3:** Pure hedonic model of milled rice price as a function of selected rice quality attributes in Bangladesh.

Log_price (BDT/kg)	Mode 1—No fixed effects			Model 2—Market fixed effects^†^		
	Coefficient	Std. Error	P-Value	Coefficient	Std. Error	P-Value^‡^
Constant	2.136	0.726	0.004***	2.536	0.657	0.000***
Broken percentage 1 (%)	0.000	0.002	0.960	0.003	0.003	0.278
Broken percentage 2 (%)	-0.002	0.001	0.043**	-0.002	0.001	0.075*
Chalk percentage (%)	-0.002	0.001	0.009***	-0.001	0.001	0.008***
Color	0.412	0.177	0.021**	0.319	0.159	0.045**
LWR	0.224	0.056	0.000***	0.230	0.042	0.000***
Homogeneity (binary)	0.154	0.019	0.000***	0.139	0.017	0.000***
Double-boil (binary)	-0.023	0.024	0.349	-0.025	0.021	0.241
Single-boil (binary)	-0.037	0.032	0.243	-0.029	0.028	0.3294
Market 2 (binary)				0.014	0.033	0.7675
Market 3 (binary)				0.054	0.037	0.143
Market 4 (binary)				-0.028	0.035	0.423
Market 5 (binary)				-0.045	0.032	0.165
Market 6 (binary)				-0.062	0.030	0.037**
Market 7 (binary)				-0.151	0.032	0.000***
Market 8 (binary)				-0.117	0.038	0.002***
Market 9 (binary)				-0.121	0.037	0.001***
Market 10 (binary)				-0.150	0.035	0.000***
Observation	300			300		
F	34.18			21.81		
Adjusted R-squared	0.340			0.461		

^†^ Market variables 2 through 10 correspond to the following neighborhoods (2) Gulshan (high income), (3) Mohammadpur (high income), (4) Farmgate/Tejgaon (middle income), (5) Mirpur (middle income), (6) Agargaon Taltola (middle income), (7) Thathari Bazar/Old Dhaka (low income), (8) Jatrabari (low income), (9) Adabor (low income), and (10) Khilgaon (low income). Dhanmondi (high income) is the benchmark market.

^‡^ ***, **, * represent 1%, 5%, and 10% significance level.

The results from both models suggest that broken percentage at a rate below 24.9 percent (variable *Broken percentage 1*) has no significant impact on rice prices, whereas it modestly but significantly (p<0.05) decreases the price of rice at rates above 24.9 percent (variable *Broken percentage 2*). To illustrate, the results from Model 1 and Model 2 (coefficient of -0.0025 and -0.0020 for *Broken percentage 2*, respectively) suggest that a 1-point increase in the broken percentage above 24.9 reduces the price of rice by 0.25 percent and 0.20 percent, respectively. The findings of this study suggest that the impact of broken rice differs depending on its level (above and below 24.9 percent), and if prices truly reflect consumer preferences, that consumers are indifferent about the amount of broken rice up to a rate of 24.9 percent. These results are in line with those by [37], who found no statistical relationship between broken rate and milled rice prices in urban and rural areas in Bangladesh.

Chalkiness was estimated to have a small but significant (p<0.01) impact on rice price in both models. The results from Model 1 and Model 2 (coefficient of -0.0016 and -0.0015 for *Chalk percentage*, respectively) suggest that a 1-point increase in the chalk percentage reduces the price of rice by 0.16 percent and 0.15 percent, respectively. These significant but marginal impacts reveal that consumers in Bangladesh are largely indifferent to chalkiness, which may be influenced by the fact that most of the rice consumed in Bangladesh is parboiled, which changes the color of rice from white to more yellow and changes the visual aspect of chalk rice. Previous research also finds a modest impact of chalkiness, namely, that a 1-percent increase in overall chalkiness reduces rice price by 0.10 in Bangladesh [16].

Color has a positive and significant (p<0.05) impact on price in both models. Since color is specified in a log form (variable *Color*), the coefficients can be interpreted as elasticities. Thus, a 1-percent increase in color results in a 0.412 percent and 0.319 percent increase in the price of rice in Model 1 and Model 2, respectively. The positive relationship between color and price is supported by the previous findings [16, 24], which show that consumers consistently rank whiteness as a valuable rice quality attribute. Nevertheless, it is interesting to note that most rice consumed in Bangladesh is parboiled, which is less white than non-parboiled rice, and despite our objective measurement, whiteness is a subjective attribute in the eyes of consumers.

The shape of rice (variable *LWR*) has a significant (p<0.001) and positive impact on rice price, increasing it by 0.224 percent and 0.230 percent for every 1-percent increase in *LWR* in Model 1 and Model 2, respectively. These findings align with previous results that suggest that Bangladeshi consumers value slenderness [16, 24], and that *LWR* has a positive and large impact on rice prices in rural and urban Bangladesh [37].

Regarding the homogeneity of rice (*Homogeneity*), the results suggest that rice mixes have a positive (0.154 and 0.139 for Model 1 and 2, respectively) and significant (p<0.001 for both models) impact on rice prices relative to pure types (e.g., long grain, medium grain, and short grain). To our knowledge, this is the first study objectively assessing this attribute, but the results are counterintuitive since we expected that rice mixes would have an inferior appearance and be priced lower than more homogeneous-appearing rice.

Finally, Model 2 shows that, all else equal, rice is priced significantly (p<0.001) lower in the four low-income markets of Thathari Bazar/Old Dhaka (variable Market 7), Jatrabari (variable Market 8), Adabor (variable Market 9), and Khilgaon (variable Market 10), than in Dhanmondi (Market 1).

### Marginal price effects

To help understand the impact of the rice quality attributes on the price of rice in Bangladesh, we estimate marginal effects using the estimates from Model 1 and Model 2 (Table 3) by estimating the rice price at the minimum and maximum attribute values observed in our study (Table 1), keeping all other independent variables at their mean values. Thus, the marginal price effect shows how much the price of rice changes with changes in the range of quality variables in the sample.

**Fig 1:**
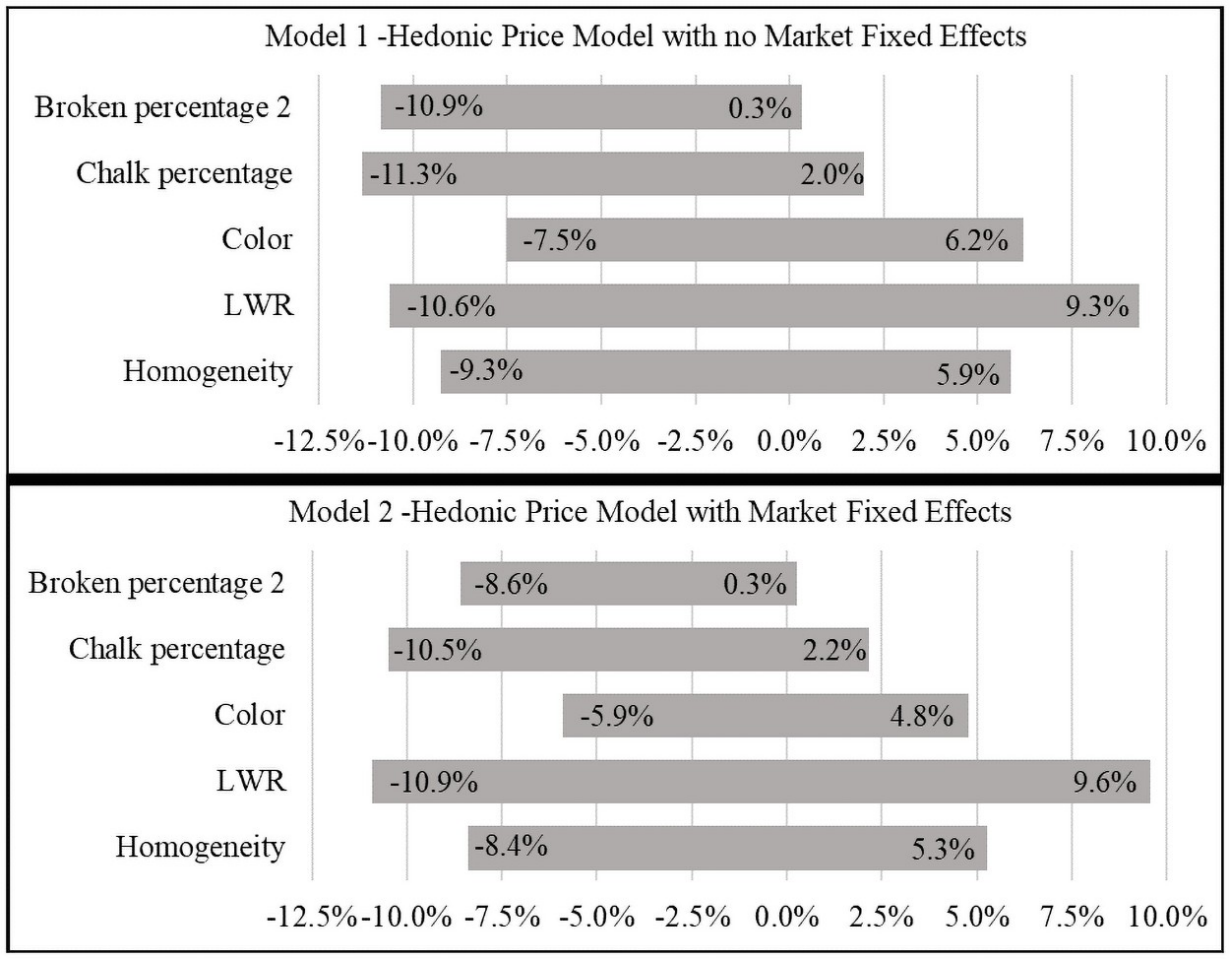
Marginal price effect of the rice quality attributes with a statistically significant effect.

Looking at the marginal effects from Model 1 (Fig 1), *LWR* has the largest marginal effect, implying that the price for rice with a low (high) *LWR* of 1.59 (3.90) is 10.6 percent lower (9.6 percent higher) than the average price. Homogeneity also has a significant (although counterintuitive) marginal effect, and requires a 9.3 percent discount if it is a homogeneous sample and a 5.9 percent premium if it is mixed relative to the average market price. Changes in the chalk percentage over the range observed in this study affect rice price from an 11.3 percent discount for rice with a chalk percentage of 89.56 to a 2.0 price premium for rice with a chalk percentage of zero. Finally, a broken percentage above 24.94 (variable *Broken percentage 2*, which has a mean value of 1.31 percent, representing a broken percentage of 26.25) can change rice price from a discount of 10.9 percent for the highest broken rate observed in this study (46.15 percent) to 0.3 percent premium for rice with exactly 24.94 percent broken rate (*Broken percentage 2* = 0).

### Food security and environmental implications of increasing the broken rate of milled rice

The fact that the broken percentage below 24.9 percent was estimated to have no impact on price, while the average broken rate in the market is 5 percent (low compared to global standards), could be seen as a lost economic opportunity, since mixing more broken rice into milled rice could increase revenues due to the fact that broken rice mixed and sold as milled rice (harmonized system (HS) code 100630) carries a higher price than if sold as 100 percent broken (HS 100640).

We claim that such quality mismatch is also a lost opportunity in the fight to improve food security in an environmentally sustainable way, because it reduces the supply of rice for food (e.g., amount of rice rations) produced per unit of input (e.g., land, fertilizer, irrigation water, etc.) and creates a surplus of broken rice used for other non-human purposes. Directing the use of broken rice for food by increasing the broken percentage of the milled rice sold in the market reduces leakages/inefficiencies (broken rice leaving the food system and used for other purposes) and improves the efficiency of producing rice rations, which leads to greater supply of rice for food per metric ton of rice.

To support the claim above, we need to first estimate the potential volume of rice suitable for quality grading based on broken percentage. Around 40 percent of the rice produced in Bangladesh is kept on-farm for self-consumption and processed using primarily home-made tools such as Dhekis [15]. The remaining 60 percent of the rice is processed by around 17,000 rice mills in operation across Bangladesh. The vast majority of these mills use outdated technologies, such as single-pass mills, which do not allow the separation of broken and whole rice. Based on the above, it is safe to assume that the broken rice produced by households and small mills using these traditional milling processes cannot be separated from whole rice, and, therefore, is already being used for human consumption. However, the opportunitiy for increasing the broken rate of milled rice is for modern mills that have the ability to separate broken and whole rice and thus control the amount of broken rice in the milled rice sold, which in the context of Bangladesh are termed as semi-automatic and automatic mills. There were 500 modern mills operating in Bangladesh in 2005 [15], and many new mills have been set up since then to attend the growing demand for milled rice. According to the Bangladeshi Rice Miller’s Association, there were 1,000 modern mills (650 semi-automatic and 350 automatic mills) in operation in 2012 [16]. Furthermore, modern mills are on average 2.5 times more productive than traditional mills [15]. With the information above, and considering Bangladesh’s average production of 51.75 MMT a year in 2016–2019 [13], we estimate that modern mills processed 4.57 MMT per year, or 8.82 percent of the annual production of paddy rice in 2016–2019.

$$\begin{array}{c} Volume\;of\;rice\;processed\;annually\;by\;modern\;mills\;=\;Average\;annual\;production\;*\;(1-share\;on-farm\;self\;consumption)\;*\;(\#\;modern\;mills/total\;\#\;mills)\;*\;productivity\;ratio\;of\;modern\;mill\;=\;51.75\;MMT\;*\;(1-0.4)\;*\;(1,000\;modern\;mills/17,000\;mills)\;*\;2.5\;=\;4.57\;MMT \end{array}$$ (vii)

We consider the quality of paddy rice in Bangladesh to be equivalent to the procurement standard used by the Food Corporation of India for “Grade A” rice in the 2020–21 *Kharif* crop [38], which reflects a paddy rice quality of 52.5/70 (52.5 percent head rice, 70 percent milled rice), and a broken percentage of 25 percent according to Eq (i). Eq (i) can be used to estimate the amount of broken rice needed to produce milled rice with a target broken rate as follows:

$$\begin{array}{c} {WB}_{i}=\frac{{WH}_{i}}{\left(1-Broken\;{percentage}_{i}\right)}-{WH}_{i} \end{array}$$ (viii)

*Assuming a 52.5/70 quality of paddy rice, 4.57 MMT of paddy rice produce 3.20 MMT of milled rice with a broken rate of 25 percent by consuming all broken rice (zero broken rice surplus), which is enough to feed 22.12 million people per year in Bangladesh at the average per-capita consumption of 144.5 kg/year* [11] (Table 4). However, the same volume (4.57 MMT) of 52.5/70 paddy rice yields 2.52 MMT of milled rice with a 5 percent broken rate, similar to that found in our sample, which is enough to feed 17.46 million Bangladeshis/year at that country’s average rice consumption rate. The production of milled rice with 5% broken rice using 4.57 MMT of 52.5/70 quality paddy rice generates a surplus of 673 thousand metric tons (TMT) of broken rice, which typically leaves the human food system and is used for feed, or even as a feedstock in the production of ethanol [39]. The findings above suggest that the production of milled rice with a 5 percent broken rate amounts to a loss of 4.66 million rice rations relative to producing milled rice with 25 percent broken. Or inversely, by increasing the amount of broken rice in milled rice from the average 5 percent observed in the market to 25 percent, the broken rate implied by a 52.5/70 paddy rice quality, we can increase the number of rations by 4.66 million a year.

**Table 4:** Volume (in thousand metric tons) of milled produced and broken rice used, rice rations produced, and harvested area saved based on the quality of milled rice produced by modern mills assuming a quality of 52.5/70.

Milled Rice Quality (% broken)	Milled Rice Production^†^	Broken Rice Use^†^	Broken Rice Surplus^†^	Rations produced (million)^‡^	Area saved^€^ (1000 has)
25%	3,196	799	0	22.12	170.79
15%	2,820	423	376	19.52	85.39
10%	2,664	266	533	18.43	42.70
5%	2,523	126	673	17.46	0.00

^†^ The production of milled rice and use of broken rice follows the assumption that modern mills process 2.28 MMT of 52.5/70 paddy rice a year.

^‡^ The rations produced are estimated using an average per-capita consumption of 144.5 kg/year [5].

^€^ The area saved is estimated as the difference between the area needed to produce same number of rations obtained with 5% broken, considering an average rice yield of 4.44 MT/hectare in 2016–2019 [3], and a milling rate of 70% as implied by a 52.5/70 paddy rice.

Another way of looking at the efficiency gain of producing rice rations (per unit of paddy rice) due to increasing the broken rate of milled rice, is by estimating the rice area saved to achieve a given number of rice rations. At the average yield of 4.44 metric tons/hectare observed in Bangladesh in 2016–2019 [13], we estimate that Bangladesh can produce 17.46 million rice rations with 25 percent broken rate annually using 170.79 thousand fewer hectares of rice compared to rice rations with 5 percent brokens.

The ability to produce the same number of rice rations with fewer production inputs improves the environmental sustainability of food security in two main ways. First, it reduces the environmental footprint of producing rice rations. Using a life cycle analysis (LCA) approach, it was estimated that the global warming potential (GWP) per kilogram of rice produced in Bangladesh across the three rice seasons (Boro, Aus, and Aman) and two technologies (traditional and high yielding varieties) ranges from a low of 1.06 to a high of 3.37 kg CO2 equivalent for Aman high yielding varieties and Aus traditional varieties, respectively [17]. We weight these GWP estimates by the rice production by season and technology [40], according to which the average 2016–2019 production share by rice season and technology was: (1) 5.01% for Aman using traditional varieties, (2) 34.07% for Aman using high-yielding varieties, (3) 0.61% for Aus using traditional varieties, (4) 6.64% for Aus using high-yielding varieties, (5) 0.19% for Boro using traditional varieties, and (6) 53.48% for Boro using high-yielding varieties. Following this approach, we estimate that a kilogram of paddy rice produced in Bangladesh in 2016–2019 has a GWP of 1.95 kg CO2 eq. Multiplying this by the volume of paddy rice saved by the change in the broken rate of milled rice from 5 percent to 25 percent (170.79 thousand hectares times the average yield of 4.44 metric tons/ha = 653.09 TMT) results in a reduction of 1.48 MMT of CO2 eq., which amounts to a decrease of 1.77 percent in the emissions generated by the agricultural sector in Bangladesh in 2016 [41].

Second, the higher efficiency in the production of rice rations lowers the pressure on natural resources, mainly arable land and fresh water, and thus counters the potentially negative impact of climate change on rice production. Bangladesh is vulnerable to climate change due to its low elevation, high population density, poor infrastructure, and its dependency on agriculture. Previous research [42] estimates that Bangladesh could lose between 0.54 percent, 1.25 percent, and 8.34 percent of the rice cropland to inundation with a 1-, 2-, and 5-meter rise in sea level, respectively. Considering the average 2016–2019 rice cropland, that amounts to 62.94, 145.67, and 972.03 thousand hectares of rice that could be loss due to a 1-, 2-, and 5-meter increase in sea level rising, respectively. Hence, the rice area saved by increasing the broken rate in milled rice could potentially offset the negative impact of a 2-meter rise in sea level on rice cropland in Bangladesh.

The implications discussed above rest on several assumptions, including the quality of milled rice produced in Bangladesh, which is assumed to be 52.5/70, and equivalent to the procurement standard used by the Food Corporation of India for “Grade A” rice in the 2020–21 *Kharif* crop [38]. Improvements in rice quality, for instance due to higher-quality improved rice varieties or other upgrades along the supply chain (e.g., better agronomic and crop management practices, improved rice drying and milling technologies) will diminish the impact of our implications. For example, Table 5 shows that if the average quality of milled rice improves from 52.5/70 to 55/70 (the commercialization standard used in the United States), the production of milled rice with a 5 percent broken rate amounts to a loss of 3.83 million rice rations relative to producing milled rice with 21.4 percent broken. In terms of rice area saved to achieve a given number of rice rations, we estimate that at the average yield of 4.44 metric tons/hectare observed in Bangladesh in 2016–2019 [13], the country can produce 18.29 million rice rations with 21.4 percent broken rate annually using 147.0 thousand fewer hectares of rice compared to rice rations with 5 percent brokens.

**Table 5:** Volume (in thousand metric tons) of milled produced and broken rice used, rice rations produced, and harvested area saved based on the milled rice produced by modern mills assuming a quality of 55/70.

Milled Rice Quality (% broken)	Milled Rice Production^†^	Broken Rice Use^†^	Broken Rice Surplus^†^	Rations produced (million)^‡^	Area saved^€^ (1000 has)
21.4%	3196	685	0	22.12	146.97
15%	2955	443	242	20.45	89.46
10%	2790	279	406	19.31	44.73
5%	2644	132	553	18.29	0.00

^†^ The production of milled rice and use of broken rice follows the assumption that modern mills process 2.28 MMT of 55/70 paddy rice a year.

^‡^ The rations produced are estimated using an average per-capita consumption of 144.5 kg/year [5].

^€^ The area saved is estimated as the difference between the area needed to produce same number of rations obtained with 5% broken, considering an average rice yield of 4.44 MT/hectare in 2016–2019 [3], and a milling rate of 70% as implied by a 55/70 paddy rice.

## Conclusion

The rice sector in Bangladesh is a major contributor to the agricultural economy, and is the main staple food for nearly every household. The dominance of rice in the agricultural production and consumption matrix heightens the importance of ensuring that markets work efficiently in the sense of pricing rice according to the quality offered so that consumers can choose the rice options that best fits their preferences and budgets, and the supply chain can properly source and generate the rice to meet that demand. This study estimates the economic value of the selected intrinsic rice quality attributes deemed of importance in Bangladesh, and the food security and environmental implications of producing milled rice with the quality characteristics indentified in this study relative to what is currently offered in the market.

These results suggest that the broken percentage, chalk percentage, LWR, color, and homogeneity of rice all have a statistically significant impact on rice prices. The shape of rice, represented by the LWR, has the largest impact on rice price, followed by homogeneity. The chalk percentage has a modest impact, while the broken percentage is statistically significant only when rates go above 24.94 percent, and even then it has only a marginal impact on rice prices.

The fact that the broken rate has such a minor impact on rice prices highlights a potentially relevant market inefficiency, because broken rice traditionally carries a price discount vis-à-vis whole rice that in this case is not being transmitted to consumers. This disconnect between brokens produced and brokens marketed is problematic from a food security perspective in two possible ways. First, it may mean that the market is overpricing rice with high percentage of broken rice to the detriment of consumers that prefer cheaper rice with higher brokens but are forced to pay a higher price for higher quality rice. Second, the striking difference between the (low) average broken percentage among the rice samples used in this study and the threshold broken percentage of 24.94 at which rice prices react to the broken percentage may mean a potential missing opportunity for selling more broken rice for human consumption.

Pricing rice accurately based on the broken percentage could improve food security by allowing consumers to either (1) afford to buy more rice with a given budget, or (2) improve the intake of other food items by buying the same amount of rice for less. Furthermore, our results suggest that there is room to increase the broken rate of the milled rice processed by modern mills, which amount to around 8.82 percent of the total rice production. We show that an increase in the broken rate from the observed 5 percent to 25 percent broken rate impied by a 52.5/70 quality paddy rice similar to the standard used in neighboring India can lead to substantial reductions in greenhose gases and help offsetting some of the potential negative impacts of climate change, such as rising sea levels.

Despite the importance of rice as a global staple, assessments of the impact of rice quality attributes on consumer demand are lacking. We argue that studies such as this offer an opportunity to ascertain how the market prices rice based on quality because the framework is easy and affordable to implement, and can provide valuable information to guide marketing and policy decisions, such as the development of grading standards, investment in production and milling technologies, and research and extension programs and policies to foster a production sector catered to the demands of the market. Importantly, this study helps fill the important void in the literature pertaining to how rice quality preferences impact regional food insecurity as well environmental suustainability.

This study has several limitations, including the lack of respondents’ socioeconomic information and the exclusion of other rice quality attributes that may drive consumers’ choices. Previous studies show that socioeconomic conditions affect consumer preferences for rice across South and Southeast Asia [9, 20], and that other attributes aside from those in this study can influence consumers’ choices (see literature review). Furthermore, we acknowledge that some of the broken rice surplus may be used as animal feed and, thus, should not be counted entirely as a loss from a human consumption point of view. However, we do not have reliable information on the use of broken rice to expand our analysis.

Notwithstanding these limitations, we believe this study shows a simple and low-cost approach to ascertain the potential inefficiencies in the rice market that could be undermining progress towards improving food security and sustainability. Methodological aspects, such as collection of socioeconomic information from consumers, selection of markets, and even quality attributes to include, must be carefully considered to make sure the results are valid for the market of interest.

## Supporting information

S1 File Bangladesh standard specification for grades on milled rice. (PDF)

S2 File Results from piecewise analysis. (PDF)

S3 File Data file. (XLSX)
