# Supplementary material for: Rice quality and its impacts on food security and sustainability in Bangladesh
Source: PLoS One. 2021 Dec 31;16(12):e0261118. doi: 10.1371/journal.pone.0261118 (PMC8719737; doi:10.1371/journal.pone.0261118)
Supplement: S1 File — (PDF) [file pone.0261118.s001.pdf]

S1 File

Bangladesh Standard Specification for Grades on Milled Rice (First Revision, BDS 592 : 1981).

Table A 1. Classification of rice varieties based on size and shape, and definition of head and broken rice.

Size
Extra long grain: ≥ 7.00 mm.
Long grain: 6.0 mm. - 6.99 mm.
Medium grain: 5.0 mm. - 5.99 mm
Short grain: < 5.0 mm.
Shape (Length/Breadth)
Slender: > 2.8
Medium: 2.1 – 2.7
Bold (Coarse): < 2.1
Definition of head and broken rice
Head rice: ≥ 8/10 of grain length
Broken rice: ≥ 1/2 of grain length
Small broken rice: ≤ 1/2 of grain length

Table A 2. White (non-parboiled) rice standard.

Item No.	Grading factor	Grading requirements			
		**Grade-I**	**Grade-II**	Grade-III	Grade-IV
1.	Moisture, percent by mass (max.)	14.0	14.0	14.0	14.0
2.	Head rice, percent (min.)	90.0	85.0	75.0	68.0
3.	Big brokens, percent (max.)	8.0	12.0	20.0	25.0
4.	Broken and small brokens, percent (max.)	2.0	3.0	5.0	7.0
5.	Damaged grain, percent (max.)	0.5	1.0	2.0	3.0
6.	Contrasting varieties, percent, (max.)	2.0	5.0	10.0	15.0
7.	Chalky/immatured grain percent, (max.)	1.0	2.0	3.0	4.0
8.	Paddy (grain per 1000 gm.)	1.0	2.0	3.0	4.0
9.	Foreign matters, percent, (max.)	0.2	0.3	0.5	1
10.	Degree of milling	Extra well milled	Well milled	Reasonably milled	Under milled

Notes: (1) Any rice not falling in any of the above grades shall be considered as sub-standard. (2) The grade requirements are expressed in percentage except for paddy seeds.

Source: Bangladesh Standards and Testing Institute, 1981.

Table A 3. Parboiled rice standard

Item No.	Grading factor	Grading requirements			
		Grade-I	Grade-II	Grade-III	Grade-IV
1.	Moisture, percent by mass (max.)	14.0	14.0	14.0	14.0
2.	Head rice, percent (min.)	95.0	90.0	85.0	80.0
3.	Big brokens, percent (max.)	4.0	8.0	12.0	16.0
4.	Brokens and small brokens, percent (min.)	1.0	2.0	3.0	4.0
5.	Damaged grain, percent (max.)	0.5	1.0	2.0	3.0
6.	Contrasting varieties, percent (max.)	2.0	5.0	10.0	15.0
7.	Paddy (grain per 1000 gm.)	1.0	2.0	3.0	4.0
8.	Foreign matters, percent (max.)	0.3	0.4	0.5	1.0
9.	Degree of milling	Extra well milled	WELL MILLED	Reasonably well milled	Under milled

Notes: (1) Any rice not falling in any of the above grades shall be considered as sub-standard. (2) The grade requirements are expressed in percentage except for paddy seeds.

Source: Bangladesh Standards and Testing Institute, 1981.
