# Supplementary material for: Rice quality and its impacts on food security and sustainability in Bangladesh
Source: PLoS One. 2021 Dec 31;16(12):e0261118. doi: 10.1371/journal.pone.0261118 (PMC8719737; doi:10.1371/journal.pone.0261118)
Supplement: S2 File — (PDF) [file pone.0261118.s002.pdf]

S2 File

Results from piecewise analysis

The steps involved in a piecewise analysis include (1) a preliminary visual assessment of the independent variable to identify a priori values for the slope and threshold point, and (2) the estimation of the regression using those a priori values to assess whether there is a statistically significant difference in the relationship between the independent and dependent variable. The piecewise analysis was performed using the NL command in Stata®.

Table B 1 shows the results for the piecewise analysis for broken rice and highlights the existence of two distinctive segments: a first segment from 0 to 24.94 broken percentage with a slope of -0.404 ($p < 0.05$) (which means that the rice price decreases by BDT 0.404/kg for every percentage increase in the broken percentage), and a second segment of the curve with a steeper slope that is not significantly different from zero ($P>0.10$). Figure B 1below shows the distribution of the original and fitted values and the threshold broken percentage at which the slope changes significantly.

Table B 1. Results from the piecewise regression analysis for broken percentage.

Price	Coefficient	Std. Error	P>t
Intercept 1	66.083	0.951	0.000***
Slope 1	-0.404	0.165	0.015***
Intercept 2	24.940	38.977	0.523
Slope 2	-0.619	0.573	0.281
Observations	300		
R-squared	0.084		

Figure B 1. Scattered plot of original and fitted values of broken percentage, and threshold of broken percentage indicating a change in slope.

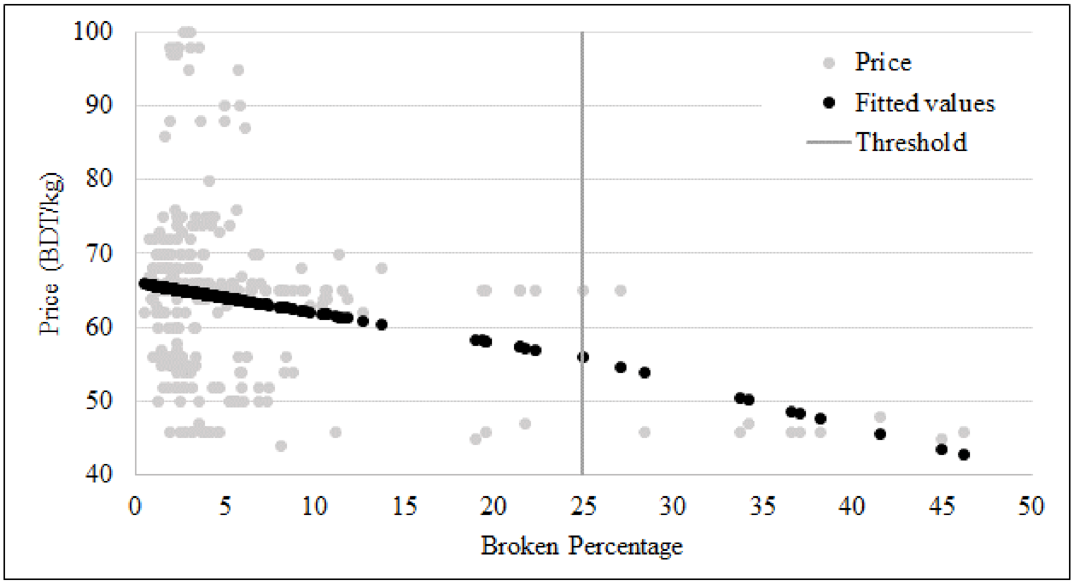

The same approach was used to ascertain whether there was a difference in the relationship between rice price and chalk percentage, and the results show that the Chalk percentage can be treated as a single variable with the same relationship to price over the range observed in the sample.
